# Anti-Peroxyl Radical Quality and Antibacterial Properties of Rooibos Infusions and Their Pure Glycosylated Polyphenolic Constituents

**DOI:** 10.3390/molecules180911264

**Published:** 2013-09-12

**Authors:** Madeline J. Simpson, Daisy Hjelmqvist, Camilo López-Alarcón, Nadja Karamehmedovic, Thomas G. Minehan, Akop Yepremyan, Baback Salehani, Eduardo Lissi, Elizabeth Joubert, Klas I. Udekwu, Emilio I. Alarcon

**Affiliations:** 1 Department of Chemistry and Centre for Catalysis Research and Innovation, University of Ottawa, Ottawa, ON K1N6N5, Canada; 2 Department of Neuroscience, Karolinska Institutet, Swedish Medical Nanoscience Centre, S-17177 Stockholm, Sweden; 3 Departamento de Farmacia, Facultad de Química, Pontificia Universidad Católica de Chile, Santiago 7820436, Chile; 4 Department of Chemistry and Biochemistry California State University Northridge, CA 91330-8262, USA; 5 Departamento de Ciencias del Ambiente, Facultad de Química y Biología, Universidad de Santiago de Chile, USACH 9170022, Chile; 6 Post-Harvest and Wine Technology Division, Agricultural Research Council (ARC), Infruitec-Nietvoorbij, Private Bag X5026, Stellenbosch 7599, South Africa; 7 Department of Food Science, Stellenbosch University, Private Bag X1, Matieland (Stellenbosch) 7602, South Africa

**Keywords:** ORAC indices, rooibos infusions, glycosylated polyphenols, bacteria, antioxidant capacity

## Abstract

The anti-peroxyl radical quality of two aqueous rooibos infusions and solutions of their most abundant glycosylated polyphenols was evaluated using pyrogallol red and fluorescein-based oxygen radical absorbance ratios. It was observed that the artificial infusions, prepared using only the most abundant polyphenols present in rooibos and at concentrations similar to those found in the natural infusions, showed greater antioxidant quality than the latter infusions, reaching values close to those reported for tea infusions. Additionally, the antimicrobial activity of the natural and artificial infusions was assessed against three species of bacteria: Gram (+) *Staphylococcus epidermidis* and *Staphylococcus aureus* and Gram (−) *Escherichia coli.* When compared to the natural infusions the artificial beverages did not demonstrate any bacterostatic/cidal activity, suggesting that the antibacterial activity of rooibos is related to compounds other than the glycosylated polyphenols employed in our study.

## 1. Introduction

Teas and herbal infusions are natural beverages which contain compounds that are of particular interest to the health sciences due to their potential *in vivo* antioxidant properties [1,2,3,4,5,6]. Thus, several methodologies have been developed to assess the antioxidant activity of such products and their main components [7,8,9,10,11,12,13,14,15]. The Oxygen Radical Absorbance Capacity (ORAC) assay [10,16] is the most commonly used method to determine the total antioxidant capacity (TAC) of food and beverages. Its usefulness to assess the health benefits of cumulative antioxidant capacity from food intake, received recent support in a cross-sectional study investigating the potential relationships of dietary TAC with adiposity, metabolic and oxidative stress markers in healthy young adults [17]. 

One variation of the ORAC assay uses nanomolar concentrations of fluorescein (ORAC-FL) as the target molecule. The decay of fluorescence is caused by peroxyl radicals generated from the thermal decomposition of 2,2'-azo-bis (2-amidinopropane) dihydrochloride (AAPH) [16]. The values measured by this ORAC assay are more related to the reaction stochiometry rather than reactivity [12,18]. Pyrogallol red (PGR), on the other hand, has also been employed as the target molecule in an ORAC-like assay (ORAC-PGR), using micromolar concentrations of the dye [10,11,12,18,19,20]. This method produces ORAC index values that describe the reactivity of the antioxidant compounds towards the AAPH-derived peroxyl radicals. As the two target molecules employed in their respective ORAC assays measure different activities it has become difficult to correlate the indices obtained from both methods with the concentrations and reactivities of the components in a complex mixture. For a more accurate index to report the “quality” of anti-peroxyl radical compounds in a given sample, it was recently proposed that the ratio of the indices (ORAC-FL/ORAC-PGR) be employed for complex mixtures. This parameter estimates the “quality” of antioxidant compounds within a given mixture, independently of their polyphenol content [12,18]. For example, when the value of the ORAC ratio is equal to unity for a given pure compound this indicates that the particular antioxidant has a similar reactivity and stoichiometry to Trolox [12,18]. Herbal tea infusions, for example, displayed ORAC ratios in the range of 0.10–0.17 [12,18].

**Scheme 1:**
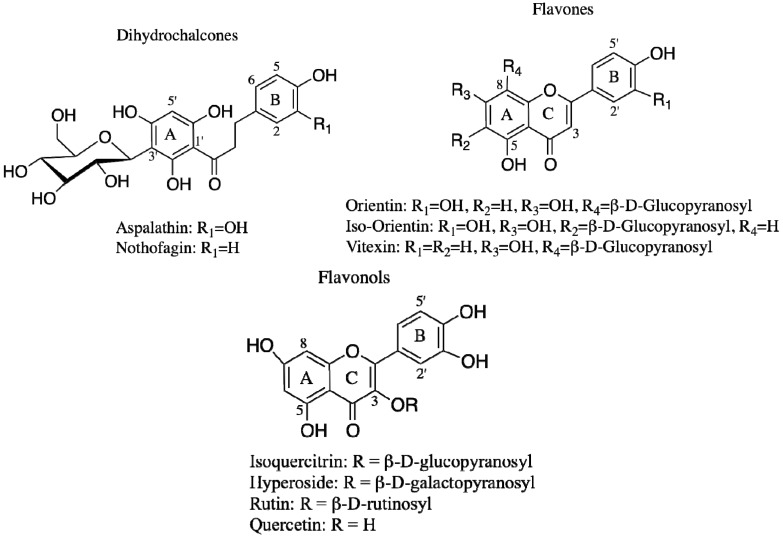
Chemical structures and atom labeling for the polyphenols of rooibos employed in this study classified according their chemical structures. Note that only aspalathin is uniquely found in rooibos.

Infusions of the herbal tea rooibos (*Aspalathus linearis*, a plant endemic to the Western Cape Province of South Africa) are caffeine-free, low-tannin beverages [21,22] that are a source of uncommon glycosylated polyphenol compounds (Scheme 1) [23,24,25]. It has been reported that the antioxidant activity of natural rooibos infusions and extracts depends on the total polyphenol content of the sample, which is determined by how the plant is processed [26,27,28]. In contrast to the polyphenol compounds present in black tea, such as the flavanols epicatechin, epicatechin gallate, epigallocatechin, and epigallocatechin gallate [1], rooibos infusions contain glycosylated polyphenols (flavonoids) including the unique dihydrochalcone, aspalathin (Asp). The anti/pro-oxidant performance of aqueous extracts of fermented and unfermented rooibos against linoleic acid and deoxyribose oxidation is directly related to their Asp content [29]. The reactivity of Asp against DPPH and superoxide anion radicals is greater than that of Trolox and similar to that of quercetin [30]. On the other hand, the ability of Asp to prevent β-carotene oxidation is considerably lower than that displayed by either α-tocopherol or quercetin [31]. The induction times become one order of magnitude larger when measured using the Rancimat method [31]. The effectiveness of these glycosylated polyphenols against reactive radical species that are directly involved in causing oxidative stress within living organisms remains unknown [32]. Recently, Joubert *et al.* [24,33] have reported the oxygen radical scavenging capacities of rooibos infusions and extracts, using fluorescence detection (ORAC-FL). However, the antioxidant quality and the contribution of the glycosylated polyphenols to that anti-peroxyl radical scavenger ability of natural rooibos infusions are still unknown. 

Rooibos extracts have also been reported to possess antimicrobial activity against *Escherichia coli*, *Staphylococcus aureus*, *Bacillus cereus*, *Listeria monocytogenes*, *Streptococcus mutans*, and *Saccharomyces cerevisiae* in liquid culture [34] and *Micrococcus luteus* and *B. cereus* growing on a solid matrix [3]. It is well known that high specificity, strong target affinity and low cytotoxicity are amongst the desirable characteristics of antibiotic compounds, as epitomized by penicillin [35]. As rooibos infusions contain naturally derived compounds they fulfill the second criterion thus making polyphenols good candidates for extraction, modification and further assessment although their specific mode of action remains unclear. Unfortunately, many other natural substances described elsewhere in the literature as antimicrobial only perform in this manner at such high concentrations that therapeutic use in their current form is precluded. 

In the present study, we have evaluated the anti-peroxyl radical quality and antimicrobial activity of green and fermented aqueous rooibos infusions and most of their abundant glycosylated polyphenols, including: aspalathin (Asp), nothofagin (Not), orientin (Ori), vitexin (Vit), iso-orientin (I-Or), quercetin-3-galactoside (hyperoside, Hyp), quercetin-3-rutinoside (rutin, Rut) and quercetin-3-glucoside (isoquercitrin, I-Qc), as well as the non-glycosylated (aglycone) flavonol, quercetin (Qc) (Scheme 1). The results were compared to those obtained using artificial infusions prepared with some of the most abundant rooibos polyphenols. Our findings indicate that Asp and Not are primarily responsible for the anti-peroxyl radical performance and quality of rooibos infusions. Artificial infusions were found to possess similar, if not superior, anti-peroxyl radical quality to the natural infusions. In stark contrast, our artificial infusions could not reproduce the growth inhibition of the clinically relevant Gram (+) bacteria *S. epidermis* and *S. aureus* or the Gram (−) *E. coli*, observed for the natural infusions*.* Our results thus indicate that the glycosylated polyphenols, although sufficient for explaining the anti-peroxyl radical activity, cannot account alone for the observed antimicrobial activity of the natural infusion. 

## 2. Results and Discussion

### 2.1. Antioxidant Performance of Pure Polyphenol Compounds and Rooibos Infusions

**Figure 1:**
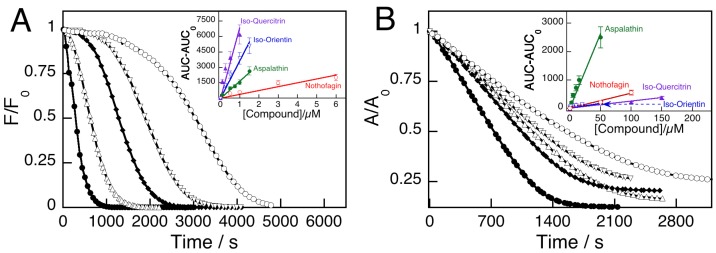
Effect of Asp on fluorescein (70 nM) decay (A) or pyrogallol red (18 µM) bleaching (B) promoted by 10 mM AAPH at 37 °C. Concentration of Asp, A: (●) control, (△) 0.1 µM; (▽) 0.5 µM; (◆) 1.0 µM and 1.5 µM (○) and B: (●) control, (◆) 2.5 µM; (△) 5.0 µM; (▽) 10 µM and 15 µM (○). Insets: Change in the area under the curve (AUC) at increasing concentrations, in µM, of selected pure polyphenols (A and B). All measurements were carried out in 75 mM phosphate buffer pH 7.4 at 37 ± 0.5 °C. ORAC indices listed in Table 1 were calculated from the linear fit of the AUC *vs.* concentration curve as shown in the insets.

The results presented in Figure 1A and B, as well as in Table 1, show that the two dihydrochalcones, Asp and Not, are the most efficient antioxidants after Qc, as demonstrated by their ORAC ratios, which are 0.11, 0.060 and 0.042 for Qc, Asp and Not, respectively. The OH groups located at C2' and C6' as well as the tautomerisation of the alpha methyl group close to the carbonyl group, are believed to play important roles in the antioxidant activity of dihydrochalcones [36,37]. The measured antioxidant activities are noteworthy because dihydrochalcones lack a suitable chemical structure, as shown in Scheme 1, to stabilize the radical formed after hydrogen abstraction from the A-ring. Kozlowski *et al.* have previously reported that an increase in the bonding dissociation energy (BDE) of the hydrogen atom of 4-OH on the A-ring for the dihydrochalcone, phloretin, modifies the reactivity of this compound towards the DPPH radical [38]. 

**Table 1:** ORAC indices for pure glycosylated polyphenols and quercetin expressed in gallic acid equivalents (GAE)/L.

Compound	Classification	ORAC-FL	ORAC-PGR	ORAC-PGR/ORAC-FL
Aspalathin	Dihydrochalcone	2.65 ± 0.13 (0.5) ^α^	0.18 ± 0.015 (5.0) ^ß^	0.068
Nothofagin		0.47 ± 0.040 (1.0)	0.020 ± 0.002 (≈25)	0.042
Orientin	Flavone	0.86 ± 0.070 (0.7)	0.0038 ± 0.0003 (≈150)	0.0044
Vitexin		4.57 ± 0.065 (0.3)	0.0078 ± 0.001 (≈55)	0.0017
Iso-orientin		4.83 ± 0.30 (0.3)	0.0077 ± 0.001 (50)	0.0015
Isoquercitrin	Flavonol	8.22 ± 0.10 (0.1)	0.0074 ± 0.0005 (50)	0.0010
Rutin		6.01 ± 0.25 *	0.009	0.0015
Hyperoside		5.50 ± 0.4 (0.1)	<0.0015 (>50)	0.0003
Quercetin		8.90 ^†^ (0.07)	1.0 ^†^ (0.5)	0.11

^†^ Average value taken from [10,15] in GAE/L. ^α^ Minimum compound concentration in µM needed to obtain induction time in fluorescein consumption. ^β^ Minimum compound concentration in µM where competition with pyrogallol red is observed. * Value taken from [16].

In addition, the OH groups on the B-ring of the dihydrochalcones could modify their antioxidant activity, as illustrated for the ORAC-PGR values of Asp and Not, with Not having *ca* one-tenth of the activity of Asp (0.118 *vs.* 0.0162). The data given in Table 1 show that the tested compounds follow the trend:

Qc ≈ I-Qc > Rut > Hyp > I-Or ≈ Vit > Asp >> Ori > Not

and:

Qc >> Asp >> Not >> Vit ≈ I-Or≈I-Qc > Ori > Rut > Hyp

for ORAC-FL and ORAC-PGR, respectively. A possible explanation for our results lies in the fact that the B-ring, particularly its 4-OH, also plays a role in the radical scavenging activity of the dihydrochalcone and the presence of a 3-OH contributes to the stabilization of the radical formed after hydrogen abstraction by a peroxyl radical. Snijman *et al.* also observed comparable antioxidant activity for Asp, Not and Qc against ABTS^+*^, while only Asp had considerable activity (0.33 times that of Qc) in inhibiting Fe(II)-induced lipid peroxidation as compared to Not (0.0125 times that of Qc). In the latter case not only radical scavenging, but other factors also played a role [23]. 

The flavone glycosides, with the exception of Ori, showed substantial ORAC-FL indices yet lower values than Qc. These glycosides had almost insignificant ORAC-PGR indices (values ranging from 3.8 to 8.0 × 10^−3^), while Qc displayed a value of 1.0. This indicates that the glycosylation of the A-ring and/or loss of the 3-OH decrease the reactivity of flavones towards peroxyl radicals.

In comparison to Qc all the 3-*O*-glycosylated quercetin derivatives tested in this study had reduced scavenger stoichiometry as demonstrated by the ORAC-FL indices listed in Table 1. The presence of the glucose moiety on I-Qc had an almost negligible effect on the ORAC-FL value measured for this flavonol when compared to its non-glycosylated parent compound Qc (8.22 *vs.* 8.9). Similarly, glycosylation of Hyp and Rut with galactose and rutinose, respectively, reduced their ORAC-FL indices by only a small percentage. These results are supported by a previous report stating that glycosylation with a monosaccharide has only a minor effect on the lipid peroxidation activity of a compound [39]. However, this apparent non-effect on the ORAC-FL index requires the assumption that this assay measures the compound’s reactivity rather than the stoichiometry of the radical trapping process (due in part to the low concentration of the target molecule, *i.e.*, 70 nM fluorescein, see [12,18]). In fact, the ORAC-PGR index was dramatically reduced after 3-OH glycosylation, *i.e.* by a factor of *ca.* 100 times for I-Qc and Rut and *ca.* 600 times for Hyp, thus agreeing with the expected decrease in reactivity due to less efficient free radical stabilization and a loss in ring co-planarity as postulated by Snijman *et al.* [23].

Two questions arise from the preceding results regarding the antioxidant activity of the pure compounds:

- Do Asp and Not control the antioxidant activity of natural rooibos infusions
- Is there a correlation between the ORAC ratios of the natural infusions with those of the artificial infusions prepared with the pure compounds in the same concentrations as present in the natural infusion

To address these questions, we present and discuss the results obtained using green and fermented rooibos infusions below, as well as two rooibos-like infusions that were prepared using most of their abundant polyphenols at the same concentrations as are found in the natural infusions (Table 2).

**Table 2:** Polyphenolic composition of green and fermented rooibos infusions. Content is expressed in g/100 g of dried infusion or as mg/mL infusion of each compound.

Compound	Green Rooibos		Fermented Rooibos	
	g/100 g dried extract	mg/mL *	g/100 g dried extract	mg/mL *
Aspalathin	10.019	0.1067	0.383	0.0031
Nothofagin	1.731	0.0184	0.151	0.0012
Orientin	0.86	0.0092	1.206	0.0097
Vitexin	0.173	0.0018	0.217	0.0017
Iso-orientin	1.068	0.0114	1.205	0.0097
Rutin	0.404	0.0043	0.064	0.0005
Hyperoside	0.217	0.0023	0.13	0.0010

* mg/mL content was calculated from the soluble solids [33], see Experimental, soluble solid values determined for green and fermented rooibos infusions were 1.0 and 0.8 mg/mL, respectively.

**Figure 2:**
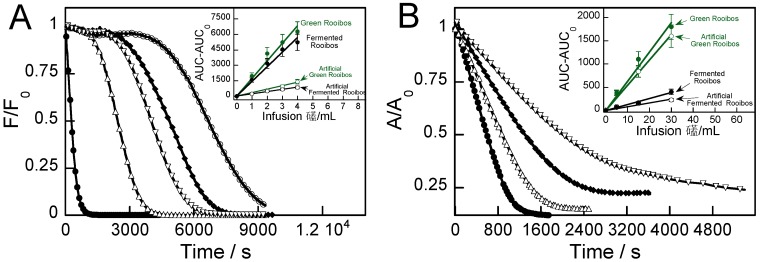
Effect of green rooibos infusion on fluorescein decay (A) or pyrogallol red bleaching (B) promoted by 10 mM AAPH thermal decomposition. Symbols correspond to artificial rooibos infusions (volume) as A: (●) control, (△) 1.0 µL/mL; (▽) 2.0 µL/mL; (◆) 3.0 µL/mL, and 4.0 µL/mL (○); B: (●) control, (△) 5.0 µL/mL; (◆) 15 µL/mL, and (▽) 30 µL/mL. Insets: Area under the curve dependence for natural or artificial (see experimental for details) rooibos infusions. All measurements were carried out in 75 mM phosphate buffer pH 7.4 at 37 °C. ORAC indices listed in Table 3 were calculated from the slope of AUC *vs.* infusion volume plots using the best linear fit.

The fermented rooibos infusion had a considerably lower Asp and Not content than the green rooibos infusion (Table 2). However, the total phenol content decreased only from 280 to 249 mg GAE/L after fermentation (Table 3). Figure 2 shows the effect of the green rooibos infusion on the decay of FL and PGR bleaching. This figure also demonstrates that addition of this natural beverage caused a time-lag prior to FL decay even at very low dilutions < 1.0 µL/mL (Figure 2A), while competition with PGR can be observed at > 5.0 µL/mL (Figure 2B). Similar effects were observed with fermented rooibos (data not shown), although fermented rooibos was at least 15% less efficient at protecting FL from peroxyl radicals (Table 3). Furthermore, the most remarkable difference between these two infusions was that the ORAC-PGR value for green rooibos was *ca.* 2.6 times greater than that of its fermented counterpart. This can be attributed to the low Asp content of fermented rooibos infusions (0.0031 mg/mL) compared to that of green rooibos infusion (0.1067 mg/mL) (Table 2). Notably, the value of the ORAC ratio calculated for green rooibos is close to the sum of the weight contribution for Asp and Not. Upon fermentation, however, the ratio resembles the value obtained for pure Not only (Table 1).

**Table 3:** Total phenol content and ORAC indices of natural and artificial rooibos.

Infusions	Phenolic content ^†^	ORAC-FL ^††^	ORAC-PGR ^††^	ORAC-PGR/ORAC-FL
Green rooibos	280 ± 20	1840 ± 168	176 ± 4.0	0.096
Fermented rooibos	249 ± 15	1520 ± 60	68 ± 5.0	0.044
Artificial green rooibos	156.9 *	600 ± 100 (0.28) ^α^	88 ± 12 (1.4) ^ß^	0.14
Artificial fermented rooibos	27.3 *	160 ± 36 (0.045)	52 ± 4.0 (0.22)	0.33

^†^ Expressed as GAE (mg gallic acid equivalents/L). ^††^ Expressed as µmol GAE/L. * Artificial infusions prepared according the composition reported in Table 1. ^α^ Minimum mixture concentration [Asp+Not] (µM) needed to obtain induction time in fluorescein decay. ^ß^ Minimum mixture concentration [Asp+Not] (µM) where competition with pyrogallol red is observed.

The results suggest that the anti-peroxyl radical quality of rooibos teas is primarily controlled by the presence of Asp and Not, which are the two most reactive polyphenol antioxidant compounds. The two artificial rooibos infusions were prepared with polyphenol concentrations chosen to imitate the composition of the natural infusions of green and fermented rooibos in terms of these dihydrochalcones and some of the other major compounds. However, the antioxidant activities of these artificial rooibos infusions were different than that of the naturally derived varieties, as seen in Table 3:

- The ORAC-FL indices of the artificial infusions were much smaller than the respective indices measured for the natural rooibos infusions;
- The ORAC-PGR indices of the artificial infusion were less than the respective indices of the natural rooibos infusions, but the differences were less pronounced than in the case of ORAC-FL.

The first observation implies that it cannot be considered *a priori* that the activity of a mixture will be equivalent to the sum of its major components [40]. In the present system, for example, the higher ORAC-FL index measured for the natural infusions can be explained in terms of the presence of poorly reactive natural antioxidants other than those considered in this study. Thus, the presence of certain antioxidants that only contribute to the protection of FL but that are not reactive enough to compete with PGR explains why only slight differences in ORAC-PGR indices are observed between the artificial and natural rooibos infusions. In addition, the considerably lower Asp and Not content of the fermented rooibos infusion is not clearly reflected in the ORAC-PGR values (Table 3). This may be interpreted as a cooperative antioxidant mechanism that occurs in the artificial infusions. However, the calculated ratio from the measured ORAC indices for the artificial samples was higher than the value for the natural beverages, where the presence of poorly reactive substances contributed, in part, to the overestimation in the ORAC-FL index with a minimal contribution to ORAC-PGR. Furthermore, the ORAC ratios measured for the artificial infusions are comparable, if not higher, to those obtained for natural infusions of black, white and green teas [18]. 

An additional fact deduced from the results of the artificial infusions, and also applicable for the natural infusions, is that only a relatively low polyphenol concentration, specifically in terms of Asp and Not, is needed to produce an observable activity, particularly for the ORAC-PGR assay. In fact, significantly lower concentrations of the artificial infusions, in the range of 0.045–0.28 µM and 0.22–1.4 µM for ORAC-FL and PGR, respectively, are needed to produce an observable effect on the consumption of the probes. These values are far lower than the required concentrations for similar ORAC assays with the pure compounds listed in Table 1. Accordingly, the most probable explanation for the increased activity is some synergetic mechanism that results in the repair of the most active antioxidants, e.g., Asp or Not, by the other compounds present in the solution, thus enhancing the antioxidant activity and decreasing the concentration needed for observable effects [41,42]. 

### 2.2. Antimicrobial Activity of Pure Compounds and Rooibos Infusions

While the antimicrobial properties of rooibos infusions and extracts have previously been described or reviewed [3,26,43], some of the polyphenols present in this herbal tea have only been shown to be active against *Helicobacter pylori* [44]. It is, however, unclear whether the antimicrobial activity of the complex rooibos mixture is due to the polyphenols alone or to some other component of the infusions and extracts. In order to ascertain this, we have compared the antimicrobial properties of natural fermented and green rooibos tea infusions with those of the artificial infusions and the individual polyphenols present in both sets of solutions. Tests were carried out on three clinically relevant species of bacteria: *E. coli, S. aureus* and *S. epidermidis*. 

**Figure 3:**
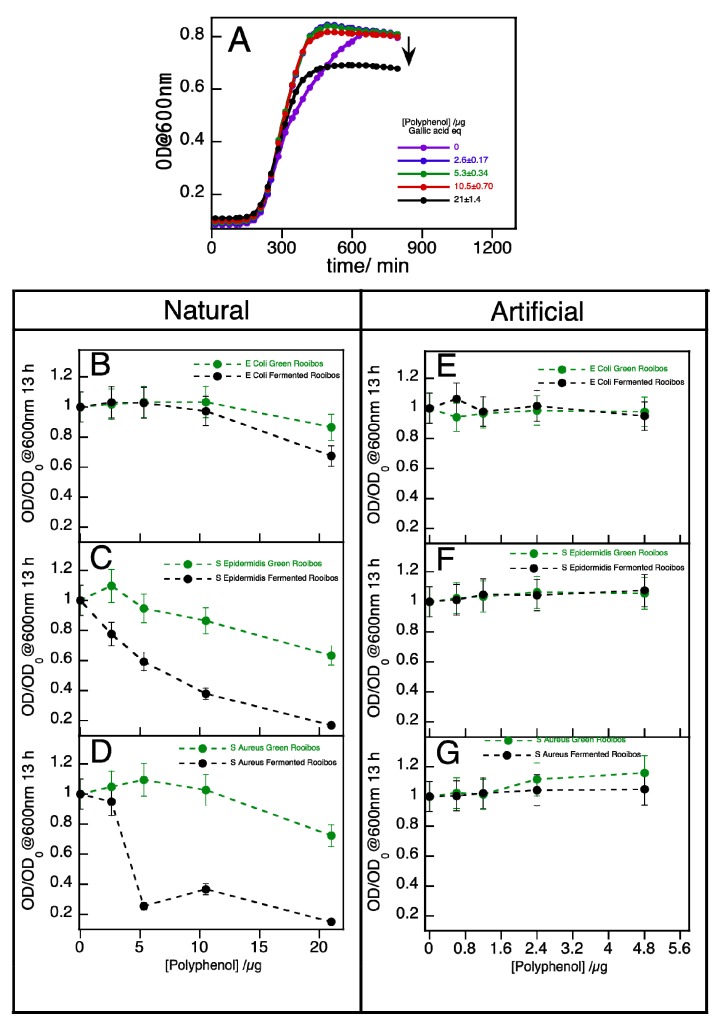
Antimicrobial efficacy of natural and artificial green and fermented infusions. Changes in optical density (OD_600_) of exposed *E. coli,* incubated at 37 °C in the presence of different dilutions (indicated as µg GAE) of natural green rooibos infusion (**A**). Variations in the OD_max_ measured at 600 nm after 13 h (expressed as OD/OD_0_), measured for *E. coli* (**B** and **E**), *S. aureus* (**C** and **F**) and *S. epidermidis* (**D** and **G**) at variable infusion concentrations of natural (**B**, **C** and **D**) or artificial (**E**, **F** and **G**) rooibos infusions. Polyphenol concentrations (for natural infusions) were estimated from the Folin-Ciocalteu polyphenol content reported in Table 3. The standard error in all cases was lower than 10% and 10% error bars have been included to facilitate data comparison.

Each bacterium species was independently exposed to variable concentrations of freshly prepared infusions of the green and fermented rooibos in Lysogeny broth (LB) over 13 h with intermittent optical density (OD_600_) estimates of growth. Figure 3A displays one representative run of the growth inhibition determination (GID) experiments for *E. coli.* The results obtained from Figure 3A were normalized to maximal optical density (OD_max_) and plotted against polyphenol concentration (µg GAE) for each of the bacterium:infusion pairs (Figure 3B–D; 3E–H). Thus, at the highest concentration of infusion tested (19 ± 1.2 and 21 ± 1.4 µg GAE for fermented and green rooibos, respectively) we observe inhibition of *E. coli* growth with the fermented rooibos infusion performing better than the green rooibos infusion (34% and 22% suppression of OD_max_, respectively, Figure 3B). 

The same trend was noted for both *S. aureus* and *S. epidermidis* with fermented rooibos outperforming green rooibos (85% *vs.* 35%, and 78% *vs.* 23%, respectively) as demonstrated in Figure 3C and Figure 3D, respectively. Overall, *S. aureus* was observed to be most susceptible to the infusions with > 70% inhibition observed for dilutions of the fermented rooibos infusion > 4.7 ± 0.3 µg GAE.

At < 4.7±0.3 µg GAE for fermented rooibos, *S. epidermidis* was inhibited by 23%. In the case of *E. coli*, the OD_max_ recorded for all concentrations below 10 µg GAE for both infusions were attained after 6 h of growth and contrary to expectation, growth was enhanced rather than suppressed. The most reasonable explanation for this is an inherent nutritional value, combined with a minimum toxicity, present at lower concentrations, similar to that observed for green rooibos at < 10 µg GAE (see Figure 3A). The results of these pharmacodynamic experiments show that at high enough concentrations, the fermented rooibos infusion possesses significant anti-staphylococcal properties. Such natural products with little or no chemical extraction requirements are thus of potential clinical benefit in low income countries and remote areas.

A relevant question is whether the observed antimicrobial activity can be attributed to the polyphenol content of the infusions alone. To address this, we exposed the same species of bacteria, in a similar fashion, to the artificial infusions, reconstituted according to the individual polyphenol content in Table 2. In this case even at the highest concentrations tested (2.0 and 4.5 µg polyphenol for fermented and green artificial infusions, respectively) no changes in bacterial growth were observed, as compared to the medium only control. This result would indicate that, at least in this system, the glycosylated polyphenols alone might not account for the inhibition observed when testing the natural extract. The reason for the discrepancy between the relative efficacies of the natural *versus* extracted polyphenols is unclear but could be due to several not necessarily mutually-exclusive reasons; bacterial uptake of polyphenols, unconsidered compounds present in the natural extracts (*vide supra*), reduced molecular efficacy, or even the presence of unidentified polyphenols with antibacterial activity. It is evident, however, that in almost all cases, the natural fermented rooibos performs significantly better than equivalent concentrations of the artificial green rooibos infusion (Figure 3). Notably, higher concentrations of quercetin in particular show significant suppression of the growth of *E. coli* and *S. aureus* (data not shown).

## 3. Experimental

### 3.1. Instruments, Chemicals and Infusions

Cary-50 UV-Visible spectrophotometer , Photon Technology International fluorometer, Agilent 1200 HPLC system. Pyrogallol red (PGR), Trolox (6-hydroxy-2,5,8-tetramethylchroman-2-carboxylic acid), gallic acid, fluorescein, and 2,2'-azo-bis(2-amidinopropane) dihydrochloride (AAPH) were purchased from Sigma–Aldrich (St. Louis, MO, USA). Folin–Ciocalteu reagent and sodium carbonate were supplied by Merck (Darmstadt, Germany). Rooibos (green and fermented) infusion bags were imported from South Africa by ZOE! Tea^®^ (Ottawa, ON, Canada).

### 3.2. Polyphenols

Orientin, vitexin, iso-orientin, isoquercitrin, hyperoside, rutin, and quercetin (> 99%) were purchased from Sigma, while aspalathin, and nothofagin were synthesized as described by Yepremyan *et al.* [45]. Both compounds were characterized by ^1^H-, ^13^C-NMR, high-resolution mass spectroscopy and IR:

*Aspalathin*: ^1^H-NMR, (400 MHz, CDCl_3_) 6.63 (d, *J* = 7.6 Hz, 1H); 6.61 (s, 1H); 6.47 (d, *J* = 8.0 Hz, 1H); 5.93 (s, 1H); 4.54 (d, *J* = 9.6 Hz, 1H); 3.87 (t, *J* = 8.4 Hz, 1H); 3.66 (d, *J* = 11.2 Hz, 1H); 3.42 (d, *J* = 8.8 Hz, 1H); 3.21 (t, *J* = 7.6 Hz, 2H); 3.14 (m, 3H) 2.70 (t, *J* = 7.6 Hz, 2H). ^13^C-NMR, (100 MHz, CDCl_3_) 204.9; 165.5; 164.4; 162.2; 145.5; 143.8; 133.0; 119.3 116.2; 115.9; 104.4; 104.1; 95.0; 81.7; 79.4; 74.1; 70.8; 70.5; 61.7; 45.8; 30.2 HRMS (ESI): Calculated for C_21_H_25_O_11_ 453.1391, found *m/z* = 453.1383 (M+H)+. IR (cm^−1^); 3368, 2925, 1626, 1527, 1454, 1372, 1283, 1115, 1080, 1041, 591.

*Nothofagin*: ^1^H-NMR, (400 MHz, CDCl_3_) 7.01 (d, *J* = 8.4 Hz, 2H); 6.65 (d, *J* = 8.0 Hz, 2H); 5.93 (s, 1H); 4.52 (d, *J* = 10.0 Hz, 1H); 3.88 (t, *J* = 8.8 Hz, 1H); 3.64 (d, *J* = 11.2 Hz, 1H); 3.42 (d, *J* = 11.2 Hz, 1H); 3.24 (t, *J* = 8.0 Hz, 2H); 3.16 (m, 2H); 2.77 (t, *J* = 7.6 Hz, 2H). ^13^C-NMR, (100 MHz, CDCl_3_) 204.8; 165.3; 164.2; 162.3; 155.8; 132.0; 129.1; 115.4; 104.4; 104.1; 95.3; 95.0; 94.3; 74.8; 73.3; 71.2; 70.3; 61.6; 46.0; 30.0. HRMS (ESI): Calculated for C_21_H_25_O_10_ 437.1442, found *m/z* = 437.1444 (M+H)^+^. IR (cm^-1^); 3341, 2925, 1627, 1515, 1541, 1367, 1245, 1171, 1080, 1018, 911, 828.

### 3.3. Infusions Preparation

Infusions were prepared by adding 150 mL of ultrapure water (95–100 °C) to a bag containing 2.0 g of material. The infusions were brewed for 5 min and then cooled to 20 °C prior to use.

### 3.4. Artificial Infusion Preparation

Artificial rooibos infusions were prepared using the most abundant and available polyphenols naturally present in this beverage based on HPLC analysis of the infusions according to the method described by [24,33]. Stock solutions (2.0 mM in pure methanol, stored at −20 °C) of the polyphenols were prepared prior to their combination in the artificial infusions, representing green and fermented rooibos. The artificial infusions were freshly prepared before each experiment. 

### 3.5. Total Phenol Content and Soluble Solids

Total phenol content of the rooibos infusions was determined using the Folin–Ciocalteu colorimetric method and gallic acid as standard [46]. For this purpose, samples (1 mL) were added to 0.2 N Folin-Ciocalteu reagent (5.0 mL). After 5 min, solid sodium carbonate (0.45 g) was added. The mixtures were incubated in the dark for 2 h at room temperature and their absorbances measured at 740 nm in a Cary 100 spectrophotometer. The sample absorbance was interpolated in a gallic acid calibration curve and expressed as mg of gallic acid equivalents (GAE)/L of infusion. The soluble solids content of the infusions was gravimetrically determined, in triplicate, using 10 mL aliquots [33].

### 3.6. Polyphenolic Content Determination

Sample analyses were performed in a similar fashion to the previously described. Briefly, freshly prepared infusions were flash frozen and lyophilized for 72 h in a Labconco Freezone 4.5 Lyophilizer. Samples were reconstituted in ultrapure water and their polyphenolic content analyzed using an Agilent 1200 system and a 100 × 4.6 mm, 1.8 µm Zorbax column protected with an Acquity UPLC in-line filter and a 5.0 µm SB-C18 guard column (Agilent) maintained at 37 °C. The flow rate was 1.0 mL/min and a multilinear gradient was performed as follows: 10% B (0–2 min), 10%–14.8% B (2–19 min), 14.8%–36.8% B (19–34 min), 36.8%–100% B (34–37 min), 100% B isocratic (37–42 min), 100–10% B (42–45 min). 

### 3.7. ORAC assays

Stock solutions of pyrogallol red (1.0 × 10^−4^ M) and fluorescein (1.0 × 10^−5^ M) were prepared daily in phosphate buffer 75 mM, pH 7.4. The consumption of pyrogallol red (5.0 µM) promoted by AAPH (10 mM) at 37 °C in the absence or presence of the tested compounds or infusions was assessed by the decrease in the absorbance measured at 540 nm (A) in a Cary 100 or SpectraMax 5 from Molecular Devices. A similar procedure was carried out using fluorescein (70 nM) as probe, but its decay was followed by the decrease in the fluorescence intensity emission (F) at 515 nm (excitation: 493 nm) in a Photon Technology International (PTI) spectrofluorometer. Indices of (F/F0) or (A/A0) were plotted as a function of time. Integration of the area under the curve (AUC) was performed up to the time when (F/F0) or (A/A0) reached a value of 0.2. These areas were employed to obtain ORAC indices, according to Equation (1) or (2) for pure compounds and complex mixtures, respectively:

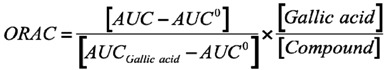
(1)

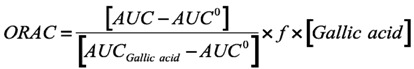
(2)
where:

AUC = area under the curve in the presence of a tested compound or infusion;
AUC_0_ = area under the curve of control experiment (without additives);
AUC_Gallic acid_ = area under the curve in the presence of gallic acid
[Gallic acid] = concentration of gallic acid expressed as mol/L,
[Compound] = concentration of tested compound expressed as mol/L, and
f = is the dilution factor, equal to the ratio between the total volume of the reaction sample and the added infusion volume.

### 3.8. Antimicrobial Activity of Rooibos Extracts and Pure Compounds

The antimicrobial activity of rooibos infusions and their pure compounds was evaluated using the Gram-negative bacterium *Escherichia coli* (CFT073) and the Gram-positive bacteria *Staphylococcus epidermis* (Strain Se19) and *Staphylococcus aureus* (Strain ATCC 25923). A growth inhibition determination (GID) protocol was employed to obtain information about the antimicrobial performance of the rooibos infusions and their main constituents. The protocol was modified [47] from the standard Clinical and Laboratory Standards Institute CLSI broth microdilution assay [48]. Briefly, exponentially growing cultures of the three test bacteria were inoculated at concentrations of ~ 5 × 10^5^ bacteria per mL, into 96 well plates containing growth medium spiked with reconstituted or natural infusions, and allowed to grow under continuous agitation over 13 h in a Biotek™ spectrophotometer set to continuously monitor OD_600_ levels every 15 min. Each experiment was carried out at ambient temperature and on duplicate samples with the assay repeated at least three independent times. Representative plots of the experiments done with *E. coli* are presented in Figure 3.

## 4. Conclusions

Our cumulative data indicate that the antioxidant activity of rooibos aqueous infusions is controlled by the presence of the two active dihydrochalcones, Asp and Not, as revealed from the ORAC ratio. We also found that only ORAC-PGR was able to measure the decrease in compound reactivity upon glycosylation as shown in the case of the quercetin glycosides, isoquercitrin and hyperoside. A synergistic effect on the antioxidant reactivity of the artificial infusions prepared using the most abundant polyphenols present in rooibos was also observed. Thus, while similar ORAC-PGR values were measured for the natural and artificial infusions, the ORAC-FL indices measured for the artificial infusions were considerable lower than for their natural counterparts. The lack of poorly reactive antioxidant molecules different from the polyphenols employed in our study is the most probable explanation for these results.

The artificial infusions did not have any significant effect on bacterial growth when compared to the natural infusions. Although the glycosylated polyphenols alone might not account for earlier observed antimicrobial inhibition in the natural extract, several possibilities may explain this. Among those delineated above, the most plausible options are either antagonism of activity (Qc showed activity in single polyphenol exposures which was not enhanced in the reconstituted infusion), or simply that other, unconsidered compounds including unidentified polyphenols are responsible. 

## Abbreviations

ORAC: oxygen radical absorbance capacity
PGR: pyrogallol red
FL: fluorescein
AUC: area under the curve
ORAC-PGR: oxygen radical absorbance capacity based on pyrogallol red consumption
ORAC-FL: oxygen radical absorbance capacity based on fluorescein consumption
ORAC ratio: ORAC-PGR/ORAC-FL
AAPH: 2,2'-azo-bis(2-amidinopropane) dihydrochloride
Asp: aspalathin
Not: nothofagin
Ori: orientin
Vit: vitexin
I-Or: iso-orientin
Hyp: hyperoside
Rut: rutin
I-Qc: isoquercitrin
Qc: quercetin
DPPH: 1,1-diphenyl-2-picrylhydrazyl radical
Trolox: 6-hydroxy-2,5,8-tetramethylchroman-2-carboxylic acid
